# An Exploratory Gene Expression Study of the Intestinal Mucosa of Patients with Non-Celiac Wheat Sensitivity

**DOI:** 10.3390/ijms21061969

**Published:** 2020-03-13

**Authors:** Konstantinos Efthymakis, Emanuela Clemente, Michele Marchioni, Marta Di Nicola, Matteo Neri, Michele Sallese

**Affiliations:** 1 Department of Medicine and Ageing Sciences, ‘G. d’Annunzio’ University of Chieti–Pescara, 66100 Chieti, Italy; efkn78@gmail.com; 2 Center for Advanced Studies and Technology (CAST), ‘G. d’Annunzio’ University of Chieti-Pescara, 66100 Chieti, Italy; e.clemente@unich.it; 3 Department of Medical, Oral and Biotechnological Sciences, ‘G. d’Annunzio’ University of Chieti–Pescara, 66100 Chieti, Italy; mic.marchioni@gmail.com (M.M.); mdinicola@unich.it (M.D.N.)

**Keywords:** wheat sensitivity, gluten sensitivity, gene expression, biomarkers

## Abstract

Non-celiac wheat sensitivity (NCWS) is a recently recognized syndrome triggered by a gluten-containing diet. The pathophysiological mechanisms engaged in NCWS are poorly understood and, in the absence of laboratory markers, the diagnosis relies only on a double-blind protocol of symptoms evaluation during a gluten challenge. We aimed to shed light on the molecular mechanisms governing this disorder and identify biomarkers helpful to the diagnosis. By a genome-wide transcriptomic analysis, we investigated gene expression profiles of the intestinal mucosa of 12 NCWS patients, as well as 7 controls. We identified 300 RNA transcripts whose expression differed between NCWS patients and controls. Only 37% of these transcripts were protein-coding RNA, whereas the remaining were non-coding RNA. Principal component analysis (PCA) and receiver operating characteristic curves showed that these microarray data are potentially useful to set apart NCWS from controls. Literature and network analyses indicated a possible implication/dysregulation of innate immune response, hedgehog pathway, and circadian rhythm in NCWS. This exploratory study indicates that NCWS can be genetically defined and gene expression profiling might be a suitable tool to support the diagnosis. The dysregulated genes suggest that NCWS may result from a deranged immune response. Furthermore, non-coding RNA might play an important role in the pathogenesis of NCWS.

## 1. Introduction

Non-celiac gluten/wheat sensitivity (NCGS/NCWS) is a syndrome dependent on gluten/wheat ingestion that presents both intestinal and extra-intestinal symptoms [1,2,3,4]. Herein, we refer to non-celiac gluten/wheat sensitivity as NCWS. This disorder was originally described in the late 1970s in patients with diarrhoea and abdominal discomfort that improved after gluten withdrawal from the diet. A more complete spectrum of NCWS clinical signs includes alternate bowel habits, bloating, abdominal pain, fatigue, foggy mind, limb numbness, dermatitis, and joint and muscle pain. These symptoms largely overlap with those of other wheat-related disorders, including celiac disease and wheat allergy [3], as well as common intestinal disorders such as irritable bowel syndrome (IBS) [5]. Given the lack of NCWS-specific biomarkers, the most advanced diagnostic algorithm implies the exclusion of confounding pathologies and the assessment of symptoms during a gluten challenge [6]. Although initially proposed as open-label [3], a more recent protocol, known as the Salerno criteria 6, entails the administration of a questionnaire where patients have to choose at least one and up to three main symptoms and give a score (ranging from 1 to 10) to their intensities during a short double-blind placebo-controlled gluten challenge. Although this protocol may be helpful in a research setting, it has never been validated clinically, nor have its positive and negative predictive values have been estimated; moreover, it may have a high nocebo effect [7], and is extremely difficult to be applied in the clinical environment, where the gluten challenge is generally administered in an open label trial. As a further limitation, this gluten-centred diagnostic protocol does not take into account other wheat components (e.g., fermentable oligo-, di-, and monosaccharides (FODMAPs) and amylase trypsin inhibitor (ATI)) that may be important in NCWS [1,2,8].

To date, we still know little about NCWS inducing factors, possible genetic predisposition, and pathological mechanisms, besides the fact that they probably differ from celiac disease and wheat allergy. As an example, the vast majority of patients with celiac disease (more than 95%) bear the human leukocyte antigen (HLA) DQ2, DQ8, or both, whereas up to 50% of NCWS patients are DQ2/DQ8-positive and the frequency of these HLA in the general population is about 30% [3,4]. Studies aimed at identifying the molecular features of NCWS reported the downregulation of forkhead box P3 (Foxp3) and the upregulation of toll-like receptor 2 and claudin-4 in the intestinal mucosa. In addition, soluble cluster of differentiation 14 (CD14), lipopolysaccharide (LPS)-binding protein, and fatty acid-binding protein 2 were shown to be increased in the plasma on these patients [9,10].

These findings suggest an involvement of innate immunity and an alteration of the intestinal permeability. Nevertheless, the intestinal mucosa of NCWS patients does not show important signs of inflammation besides a modest production of interferon gamma (IFNγ) and an increase of intraepithelial lymphocytes [4,11]. A more recent study reported a significant increase of eosinophils and clustering of T cells in the duodenal and rectal mucosa of NCWS patients as compared to controls [12], further suggesting an impact of an inflammatory immune response in NCWS.

Because NCWS presents minor signs of intestinal involvement, at least by using the classical approaches, we hypothesised that subtle changes of multiple genes might be sufficient to cause intestinal discomfort. In the present study, we tested the main hypothesis that there is a difference in gene expression between NCWS patients and non-NCWS patients. Moreover, we aimed to shed light on the molecular mechanism that regulates this disorder and to identify possible biomarkers of NCWS status that could be helpful to the diagnosis.

Therefore, we carried out a genome-wide expression analysis on RNA extracted from intestinal mucosa of 12 NCWS patients, and 7 age-matched controls.

## 2. Results

### 2.1. The Intestinal Mucosa of NCWS Patients Showed a Gene Expression Pattern Different from Controls

Total RNA isolated from the duodenal biopsies of 7 controls and 12 NCWS patients (Table 1) was used to assess gene expression by microarray analysis. Gene expression levels, measured as described in the methods, were subjected to quantile normalization and log_2_ transformation before statistical analysis. Contextual to the normalization, we excluded aberrant/unreliable probes signals, resulting in 53,218 measurements. About half of the probes targeted protein-coding transcripts (52.4%) and the other half targeted non-coding transcripts (47.6%).

We considered as non-coding RNAs the 25,334 transcripts whose gene identifiers (IDs) or symbols start with LNC, LOC, XLOC, Linc, SNORA (small nucleolar RNA), or SNORD (small nucleolar RNAs, C/D box), or end with IT (intronic transcripts) or AS (antisense RNA), as well as all the Agilent probes without a gene symbol (Table 2). This last group of 8115 transcripts were included here because a manual check revealed that, although heterogeneous, the vast majority of them are non-coding RNA. Only 303 of these non-coding RNAs (i.e., SNORA and SNORD) were short non-coding RNAs, whereas the remaining 25,031 (Table 2) belong to the class of long non-coding RNA (lncRNA).

**Figure 1:**
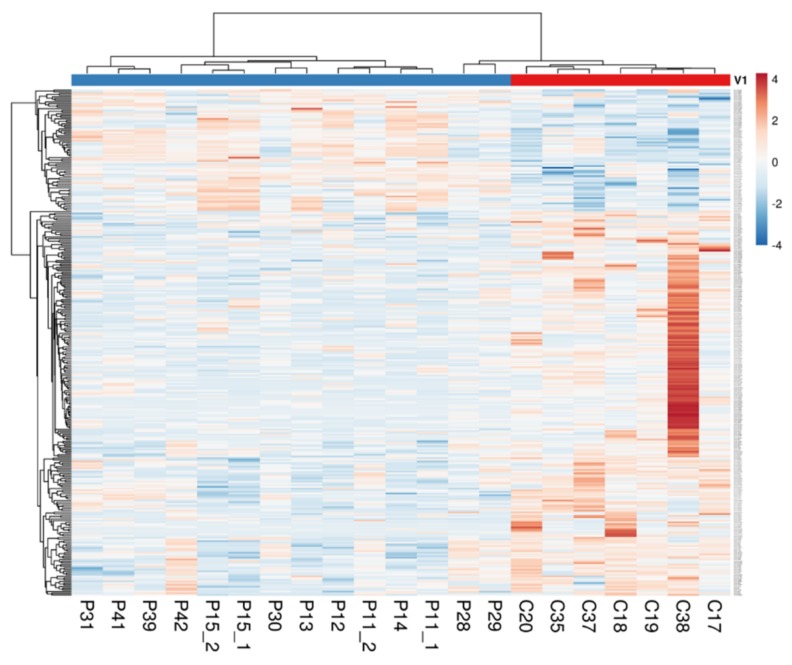
Heat map representation of the DE transcripts between NCWS and controls. Reddish and bluish colours represent upregulated and downregulated transcripts, respectively. The columns of the heat map represent the patients organised according to Canberra distance algorithm, whereas the rows represent the transcripts organised according to correlation distance. The code shown below the columns identifies a specific patient.

The differentially expressed (DE) transcripts were identified through two subsequent steps; firstly, we selected the transcripts whose mean expression differed from at least 1 unit from the mean expression of controls. The frequency distribution of these differences throughout the transcriptome (Figure S1) indicated that only a small pool of transcripts fulfilled our filtering criteria. Secondly, on the filtered transcripts, we performed a Student’s *t*-test and Benjamini–Hochberg correction (false discovery rate, FDR = 5%). This approach revealed 300 DE transcripts, of which 228 (76.0%) were significantly down-regulated and 72 (24.0%) were significantly up regulated (Table S1). Remarkably, only 36.7% of the DE transcripts were protein-coding RNA (Table 2). This bias against non-coding RNAs was even more striking when we calculated the percentage of DE transcripts within the subsets of protein-coding RNAs (0.35%) and non-coding RNAs (0.79%). Hierarchical clustering of patients and DE transcripts, shown in the heat map of Figure 1, provided a first qualitative overview about the genetic differences between NCWS and controls.

Furthermore, we used the STRING database (https://string-db.org/ [13]) to evaluate the biological connectivity of the DE transcripts. STRING contains 96 of the DE transcripts identified in this study and shows 25 connections among them, whereas by chance we would have expected only 11 connections. This enrichment was statistically significant (protein–protein interaction enrichment p-value: 0.000147) and suggested that the DE transcripts are, at least partially, biologically connected as a group [13].

### 2.2. Gene Expression can Contribute to Identify the NCWS Status

**Figure 2:**
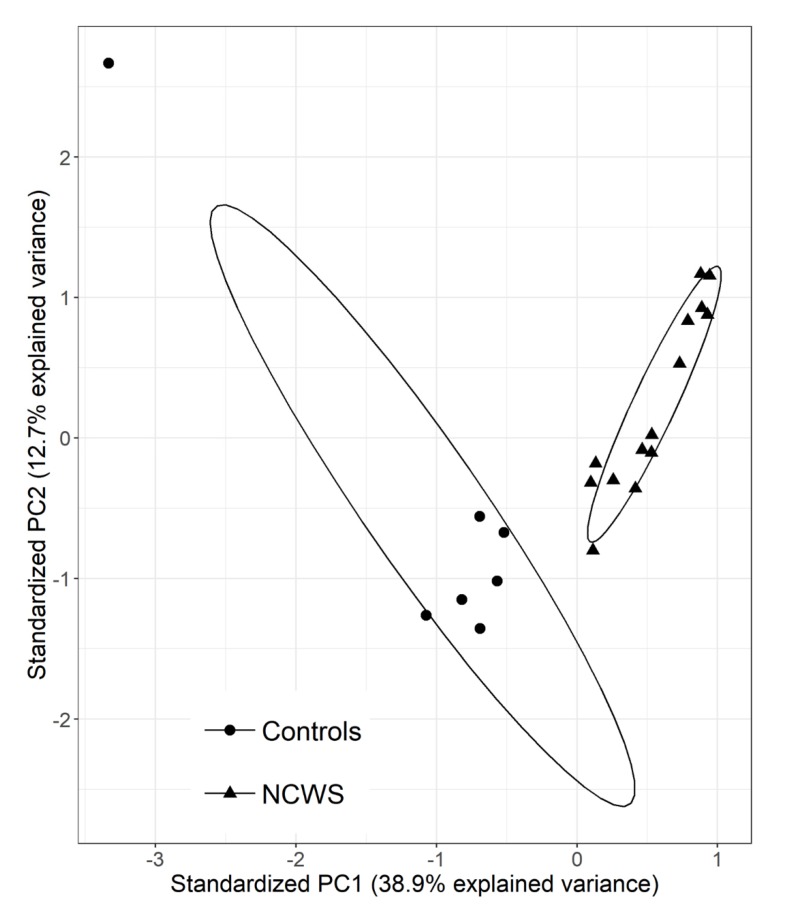
Principal component analysis of the DE transcripts. Scatter plot of the first two components (principal component (PC)1 and PC2) of the DE expressed transcripts between NCWS and controls with 95% confidence ellipses. **▲:** NCWS, and •: controls.

To reduce the number of variables (i.e., DE transcripts) and examine the relationship between gene expression and NCWS, we analysed the microarray data by the unsupervised principal component analysis (Figure 2). The first component (principal component (PC)1) explained almost 38.9% of the variance and was associated with NCWS status, whereas PC2 explained 12.7% of variance (Figure 2) and was not associated with the pathology. The new principal component analysis (PCA)-derived variable (PC1) was able to cluster samples into two groups, showing that distinct gene expression patterns were present in NCWS and controls.

**Figure 3:**
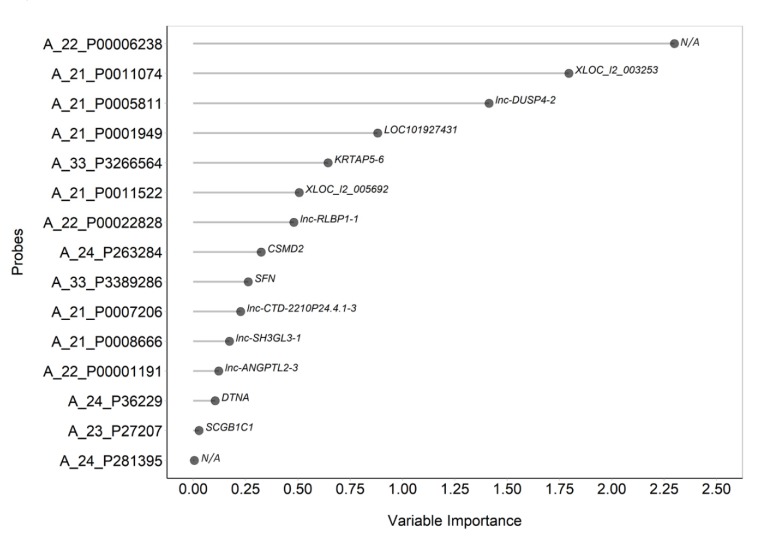
Variable importance coefficients derived from least absolute shrinkage and selection operator (LASSO) penalized logistic regression used to identify DE transcript predictive of the diagnosis of NCWS.

In order to define which transcript may contribute more to discriminate the NCWS status and if a limited set of transcripts might be sufficient for this classification, we relied on a penalized logistic regression using the LASSO (least absolute shrinkage and selection operator) method. This analysis of variables’ importance identified 15 transcripts that mainly contributed to characterisation of NCWS patients (Figure 3; Table 3). The heat map of these 15 transcripts clearly showed the different expression profiles between NCWS and controls groups (Figure S2A). The PCA of these 15 transcripts showed that NCWS patients were fairly homogeneous and this small gene expression signature had the potential to group patients and controls separately (Figure S2B). Statistical analysis of the receiver operating characteristic (ROC) area under the curve (AUC) built with the transcripts selected by LASSO indicated that indeed one transcript would be sufficient to classify NCWS patients with very high confidence (Table 3).

### 2.3. Possible Pathological Mechanisms Engaged in NCWS

**Figure 4:**
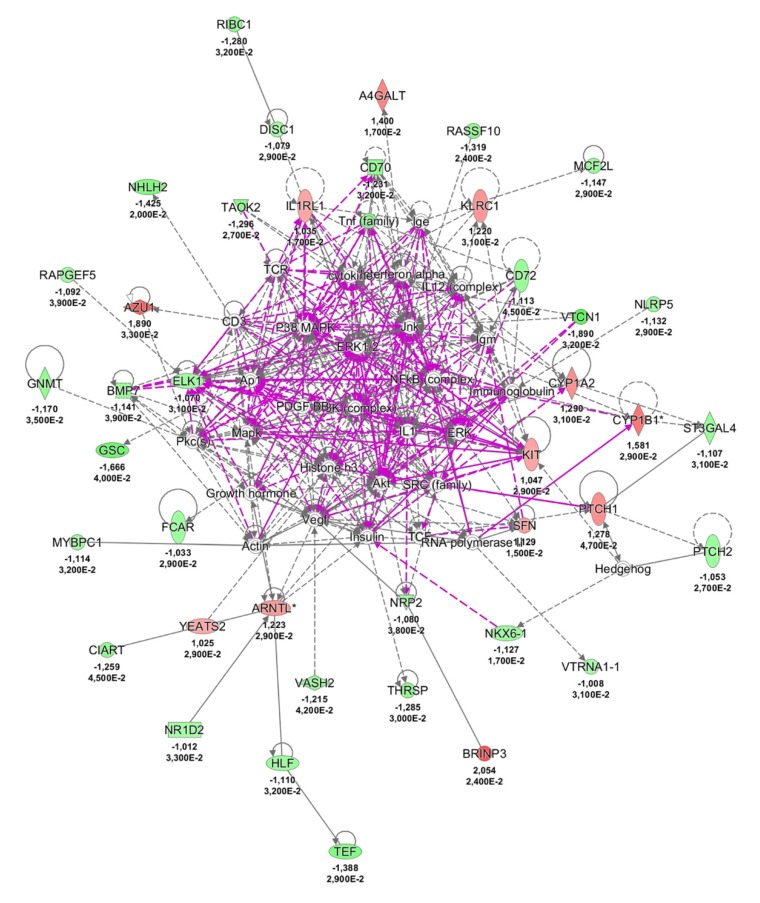
Ingenuity pathway analysis (IPA) networks associated with NCWS status. Transcripts are indicated by gene names, whereas shapes indicate gene functions. Lines connecting the nodes indicate known relationships between genes. Green and red colours indicate the up- and down-regulated genes, respectively.

Finally, to shed light on the pathological mechanisms of NCWS, we analysed the literature of DE transcripts and investigated their belonging to functional pathways by using ingenuity pathway analysis (IPA). Although most of the DE transcripts were uncharacterised or poorly investigated non-coding RNA, we obtained a network of proteins (Figure 4) suggestive of a possible role of immune response (e.g., AZU1, BMP7, CD70, CD72, FCAR, HLF, IL1RL1, KIT, killer cell lectin-like receptor C1 (KLRC1), MCF2L, MYBPC1, NOD-like receptor protein 5 (NLRP5), NR1D2, NRAP, NRP2, SFN, TAOK2, VTCN1), hedgehog pathway (NKX6-1, PTCH1, PTCH2, SFN, ST3GAL4), and regulators of circadian rhythm (ARNTL, CIART, HLF, NR1D2, TEF, YEATS2) in NCWS. Literature analysis further indicated an involvement of innate immune response and potentially of autoimmunity in NCWS; details about this claim are in the Discussion section. It is worthwhile to mention that the vast majority of the DE transcripts identified in this study had previously been reported in malignancies of the intestinal tract (Figure S3). This could simply reflect the high number of studies focusing on cancers and/or the huge amounts of molecular pathways associated with cancers (cell transformation, growth, invasion, etc.) [13].

## 3. Discussion

In susceptible individuals, the simple contact, inhalation, or ingestion of wheat derivatives can cause allergic, autoimmune, or poorly defined pathological reactions. Wheat allergy includes immunoglobulin E (IgE) immuno-mediated symptoms such as respiratory allergy, contact urticaria, food allergy, and wheat-dependent exercise-induced anaphylaxis [3,14]. Celiac disease bears a number of intestinal, systemic, and autoimmune manifestations [15]. Both pathologies rely on clinical, laboratory, and histological diagnostic criteria. On the contrary, the clinical manifestations and the pathogenic mechanisms of the recently defined NCWS are still little understood and the diagnosis is not supported by laboratory tests [6,10]. Currently, NCWS diagnosis relies on the association of gluten ingestion with symptom severity in individuals where celiac disease has been excluded by the absence of serological markers (e.g., anti-TGA and anti-EMA) [6].

We decided to investigate gene expression profiles of NCWS with the aim of shedding light on the molecular mechanisms governing this pathology and eventually identifying effective biomarkers helpful to the diagnosis. Previous studies have demonstrated that gene expression profiles have the necessary qualification to carry on this endeavour [16,17].

NCWS patients recruited for this study were middle-aged females with normal body mass index and without obvious signs of systemic or intestinal inflammation. These patients reported intestinal symptoms (e.g., abdominal pain, bloating, diarrhoea, and constipation) and a variety of extra-intestinal manifestations (e.g., fatigue, headache, brain fog, and numbness in the limbs) that worsened upon gluten ingestion. Controls did not exhibit the typical symptoms of NCWS, whereas many general diagnostic tests (e.g., erythrocyte sedimentation rate (ESR), C-reactive protein (CRP), calprotectin, and haemoglobin (Hb)) overlapped those of NCWS patients. Celiac disease was excluded by assessing specific serum biomarkers (e.g., anti-TGA and anti-EMA) and by histological evaluation of the duodenal mucosa.

The microarray platform used in this study contained about 53,000 probes targeting both protein coding and non-coding RNAs in roughly equal amounts. In the intestinal mucosa of patients affected by NCWS, we identified 300 transcripts whose expression differed from controls. Remarkably, a minor fraction of these DE transcripts were protein-coding RNAs. The remainder of the DE transcripts were lncRNA or uncharacterised RNA. Non-coding RNAs ranked in first position among those with higher discrimination power between NCWS and control groups (LASSO analysis), further underscoring the importance of this class of transcripts in NCWS.

lncRNA is a large and novel class of RNA that is still little understood [18]. They can be sub-classified into intronic lncRNAs, bidirectional lncRNAs, enhancer RNAs (eRNAs), and intergenic lncRNAs on the basis of the region of the genome that is transcribed or on the transcription mechanism [19,20]. The introduction of next generation sequencing led to the discovery of thousands of new lncRNAs in mammalian cells [18]. The functional roles and the specific mechanisms of action have been reported for various lncRNAs; nevertheless, some researchers sustain that lncRNAs are produced by spurious transcription and therefore mainly without functions [21,22]. In contrast, accumulating evidence shows that transcription of these RNAs requires transcription factors/promoters, in analogy to protein coding RNA, and their expression is regulated by cell needs and in response to pathological conditions. For example, lncRNA can control chromatin structure and gene expression, splicing machinery, the stability of mRNA, and the availability of miRNA [22]. These activities are accomplished by generating ribonucleoprotein complexes or by interacting with other nucleic acids of the cells through base pairing. From the pathophysiological standpoint, lncRNA are involved, among others, in inflammation and immune response [23]. For example, it has been reported that LPS influences the expression of 221 lncRNAs in monocytes [24]. The lncRNA tumor necrosis factor alpha-induced protein 3 (TNFaip3) works in concert with nuclear factor kappa B (NF-ĸB), whereas Lethe inhibits NF-ĸB-dependent gene expression downstream to tumor necrosis factor alpha (TNFα) [25,26]. Even cyclooxygenase-2 (COX2) expression is regulated by an lncRNA called p50-associated COX-2 extragenic RNA (PACER) [27]. In summary, lncRNAs appear to have important regulatory roles, but it is difficult to anticipate the effects of lncRNA dysregulation in NCWS. Further studies are needed to even understand whether changes in transcript expression the cause or the consequence of the disease are.

Literature analysis of the DE protein-coding RNAs identified in this study provided some insights about the pathological mechanism of NCWS. For example, ARNTL and NR1D2, two circadian clock genes, regulate inflammatory responses in macrophages by inhibiting NF-ĸB [28]. Recent studies demonstrated the importance of daily genes in the control of innate immunity [29]. NLRP5, NOD-like receptor protein 5, belongs to the superfamily of pattern-recognition receptors such as the toll-like receptors. The NOD-like receptors have an important role in innate immune response and autoimmune diseases including Crohn’s disease [30]. FCAR encodes the receptor for the crystallisable fragment of IgA, a transmembrane protein expressed on the plasma membrane of neutrophils and macrophages [31]. Previous studies demonstrated that the signalling triggered by FCAR upon binding with IgA negatively regulates the inflammatory response [32]. Patients with IgA deficit are prone to autoimmune disease, including celiac disease, in line with the inhibitory role of FCAR and IgA in immune response [33]. Therefore, the downregulation of FCAR reported in this study may indicate that NCWS shares some pathological features with autoimmune diseases. AZU1 encodes an antimicrobial protein named cationic antimicrobial protein 37 kDa (CAP37) or azurocidin 1 because of its presence inside the azurophilic granules of neutrophils. This protein participates in innate antimicrobial response and monocytes chemo-attraction [34]. KLRC1 (killer cell lectin-like receptor C1) encodes the natural killer receptor G2A (NKG2A) protein, an integral membrane receptor of the natural killer lymphocytes, key cells at the edge of innate and adaptive immune response. Previous studies demonstrated that NKG2A may participate in autoimmune diseases including psoriasis, rheumatoid arthritis, and ankylosing spondylitis [35,36,37]. BMP7 encodes the bone morphogenetic protein-7, a transforming growth factor-beta (TGF-β) family member that is able to polarize monocyte into M2 macrophage and increase the expression of anti-inflammatory cytokines [38]. We can hypothesise that BMP7 downregulation may contribute to the development of a subtle inflammation in NCWS. Indeed, previous studies have underscored the anti-inflammatory effect of BMP7 in the gut [39]. Notably, BRINP3 (bone morphogenetic protein/retinoic acid inducible neural-specific 3), another component of the bone morphogenetic protein family identified in this study, was reported as being downregulated in the intestinal mucosa of patients affected by ulcerative colitis [40]. CD70 is present on antigen-presenting cells and was found to be involved in T-lymphocyte activation, as well as in pathophysiology of autoimmune diseases including lupus erythematosus and rheumatoid arthritis [41]. CD72 is a negative regulator of B cells that is also involved in the development of lupus erythematosus via toll-like receptor 7 [42]. Glycine *N*-methyltransferase (GNMT) encodes a tumour suppressor gene recently associated with T cell immune response. In a mouse model of autoimmune encephalomyelitis, the knock down of GNMT reduced the severity of the disease [43]. CUB and sushi multiple domains 1 (CSMD1) is a complement control-related gene that inhibits the activation of complement C3 and has a possible role in lupus erythematosus and schizophrenia [44]. The RNA transcript HLA-DRB6 is considered a pseudogene; nevertheless, its expression correlates with autoimmune diseases such as rheumatoid arthritis and type I diabetes mellitus (https://www.ebi.ac.uk/gwas/search?query=HLA-DRB6).

Besides immunity, ingenuity pathway analysis (IPA) suggested that the hedgehog pathway might have a role in NCWS. It is worthwhile to mention that a few studies demonstrated the involvement of hedgehog pathway in celiac disease and alteration of immune surveillance in cancer development [45,46,47].

Our study is not devoid of limitations. First, although our data have extensive statistical validation, they are representative of a small number of patients. Second, our findings may not be applicable to patients with different characteristics because the results have not been validated in an external cohort. Finally, the diagnosis was based on an open gluten challenge. Therefore, prospective studies should be conducted on larger cohorts of patients diagnosed according to the Salerno Experts’ Criteria.

## 4. Material and Methods

### 4.1. Patient’s Recruitment

Patients referred to the Regional Centre for Adult Celiac Disease and the Gastroenterology and Endoscopy Unit at the “SS. Annunziata” University Hospital of Chieti claiming gluten-related symptoms were examined according to the current clinical practice by an expert gastroenterologist according to the established criteria at the time of the study design [3]. Those presenting with gastrointestinal symptoms, a negative serology for celiac disease (IgA anti-TGA and/or anti-EMA), a preserved mucosal architecture (Marsh grade ≤1), and a normal titre of total IgA were enrolled as potential NCWS. Subjects were also required to have negative immuno-allergy tests to wheat and a negative glucose hydrogen breath test to exclude small intestinal bacterial overgrowth (SIBO). Furthermore, patients with HLA-DQ2/8 positivity were purposefully excluded from the NCWS group when a positive family history for celiac disease among first degree relatives was reported. To the remaining potential NCWS, an experienced nutritionist prescribed an open gluten-free diet for a 6 week period, after which persistently symptom-free subjects were reintroduced to dietary wheat protein (equivalent to 10 grams of gluten). At that point, the recurrence of symptoms, together with all other clinical and diagnostic data, evaluated by an expert gastroenterologist, prompted the final diagnosis of non-celiac wheat sensitivity [3,9,48]. This was done according to the diagnostic criteria set forth at the time of the study design [3]. Symptom severity was assessed using a modified diagnostic questionnaire (Gastrointestinal Symptom Rating Scale) at baseline and after gluten exclusion.

As controls, we selected patients with dyspeptic symptoms who were prescribed endoscopic examination and reported no association of symptoms to specific dietary components at initial inclusion. Dyspepsia was defined according to the Rome III criteria. Those who accepted an elimination diet as an initial treatment strategy were prescribed a gluten-free dietary scheme by an experienced nutritionist for 4 weeks. Finally, controls were enrolled among the non-responders to a gluten-free diet, who had a normal duodenal mucosa at endoscopy and were not affected by SIBO (Table 1). Note that a few controls and NCWS patients were positive for *Helicobacter pylori* (Table 1); however, they did not carry present or past major *Helicobacter pylori*-related diseases and its presence was equally distributed between groups.

All the controls and patients underwent upper endoscopy within 1 month from laboratory assessment. At least five duodenal biopsies were obtained for histological examination, including one in the duodenal bulb. Narrow band imaging and white light magnification were used to aid biopsy sampling. Biopsy samples were placed on cellulose paper to maintain orientation and prevent artefacts. An additional biopsy destined for inclusion in this study was taken from the immediate vicinity of those intended for histological examination. Biopsies used in the study were obtained prior to any gluten exclusion diet.

NCWS duodenal mucosa, examined by histological staining, scored grade 0 or I of the Marsh scale indicating no alteration or a minor infiltration of intraepithelial lymphocytes (Table 1). Alterations of the intestinal mucosa, such as those observed in celiac disease, may cause malabsorption of nutrients, especially iron; however, ferritin and the haemoglobin levels suggested that intestinal absorption of iron was appropriate in these patients. Finally, the frequencies of HLA DQ2/Q8 were about 30%, in line with the distribution of these haplotypes in the general population.

NCWS and controls were matched for age, sex and body mass index, as well as showing comparable levels of C-reactive protein, erythrocyte sedimentation rate, haemoglobin, calprotectin, and ferritin (Table 1). These indices, although not exhaustive, suggest the absence of chronic inflammatory conditions. On the basis of their body mass index, patients were of normal weight or slightly overweight.

Ethical approval was obtained from the Clinical Research Ethics Committee dell’Università degli Studi “G. D’Annunzio” and Azienda Sanitaria Locale (ASL) N°2 Lanciano-Vasto-Chieti; report number 13, 07/18/2013. Furthermore, written informed consent was obtained from all the participants.

### 4.2. RNA Isolation and Microarray Analysis

Patients’ biopsies were collected from the duodenum near the site used for histopathological examination, immediately submerged in RNAlater and stored at 4 °C. Total RNA was extracted using the miRNeasy kit (Qiagen), quantified, and analysed by gel electrophoresis and Bioanalyzer (Agilent). All the samples used for microarray hybridization had an RNA integrity number >4. RNA samples were extracted along a 2 year period and microarray hybridization was performed on two different chip/experiments. Chip1 included the following NCWS patients: 11_1, 12, 13, 14, 15_1, and a technical replicate 15_2. Chip2 included the following controls: 35, 37, 38, 17, 18, 19, and 20; NCWS patients 28, 29, 30, 31, 39, 41, and 42; and a technical replicate 11_2 already evaluated on chip1. Therefore, a total of 7 controls, 12 NCWS, and 2 technical replicates of NCWS were analysed by microarray.

The microarray, outsourced to “Consorzio Futuro in Ricerca”, previously known as Tecnopolo Ferrara, was carried out with the Agilent Technologies. Data transformation was applied to set all the negative raw values at 1.0, followed by quantile normalization and log_2_ transformation.

### 4.3. Statistical Analysis

In order to forestall the minimal number of patients to be recruited in the study, we assumed a common difference in mean expression between the two groups equal or greater than 1 in absolute values and a common standard deviation equal to 0.5. On the basis of these assumptions, we performed a simulation of sample size estimation considering different proportions of differentially expressed genes. This simulation showed that a number of patients of 7-11 was necessary to obtain a statistical power of 80% with a false discovery rate (FDR) of 0.05. Considering the fact that the proportion of differential expressed genes in our cohort was equal to 0.7, we had approximately 85% statistical power.

Qualitative variables were summarized as frequencies and percentages, and values of continuous variables, such as gene expression, were tested for normal distribution with Shapiro–Wilk’s test and reported as mean and standard deviation (SD). The results were reported separately for each group. Mann–Whitney U test and Pearson’s chi-squared test were applied to evaluate the differences in quantitative and qualitative characteristics among groups, respectively.

To identify differentially expressed (DE) transcripts, absolute difference between mean expression in NCWS patients and controls were calculated and the transcripts with value > 1 were selected. Student’s *t*-test was applied to select transcripts with statistically significant difference between mean expression of NCWS patients and controls using the threshold FDR  ≤  0.05 [49].

An unsupervised principal component analysis (PCA) was applied to reduce the dimensionality of a microarray dataset into two components, principal component (PC) 1 and PC2. PCA was conducted as an “unsupervised” analysis to clarify the variance among microarray data from two groups using an R function “prcomp”. The proportion of variance was also calculated to determine the percentage of variance explained by each PC.

Least absolute shrinkage and selection operator (LASSO) penalized logistic regression was used to identify DE transcript predictive of the diagnosis of NCWS. LASSO model allows the selection of variables with a highest predictive ability in the situation where there is a large initial set of variables. The final values used for the logistic regression model were alpha = 1 and lambda = 0.0001 (kappa = 0.492, accuracy = 0.783). LASSO regression selected variables through shrinking the beta coefficients of unimportant variables to zero. The degree of shrinkage is determined by a penalty parameter (lambda), the value of which is identified through cross validation (10-fold, repeated five times) to select the set of variables that maximize the area under the curve (AUC).

Finally, the ability of each selected DE transcripts to predict the status of NCWS was performed using a ROC curve. The AUC was calculated as a measure of classification model performance.

DE transcript was imported into ingenuity pathway analysis (IPA, Qiagen) to generate enriched pathways and networks.

For all tests, the threshold for statistical significance was set at *p* < 0.05. All analyses were performed with the open-source statistical R software (version 3.4.3, The R Foundation for Statistical Computing).

## 5. Conclusions

This study indicated that NCWS can be genetically defined and gene expression profiling could be a suitable tool to support the diagnosis. The functional role of the dysregulated genes suggested that NCWS may result from a pathological immune response, especially of the innate branch. Furthermore, non-coding RNA could play an important role in the pathogenesis of NCWS.

## Figures and Tables

**Table 1:** Patients’ characteristics.

Variables	CTRL	NCWS	*p*-Value ^a^
Number of subjects	7	12	
Age (years), mean ± SD	56.3 ± 22.8	46.5 ± 11.7	0.228
Gender (M/F)	1/6	0/12	0.779 ^b^
Marsh classification, *n* (%)			-
0		6 (50.0)	
1		6 (50.0)	
3a		-	
3b		-	
3c		-	
TGA-IgA, (U/mL), mean ± SD	1.8 ± 1.2	2.5 ± 1.6	0.331
Hp positive, *n* (%)	4 (57.1)	2 (16.7)	0.187 ^b^
HLA-DQ, *n* (%)			0.767 ^b^
Negative	5 (71.4)	8 (66.7)	
DQ2	2 (28.6)	4 (33.3)	
EMA positive	-	-	-
ESR (mm/h), mean ± SD	12.6 ± 4.3	14.3 ± 4.7	0.444
CRP (mg/L), mean ± SD	0.4 ± 0.2	0.4 ± 0.3	0.998
Calprotectin (µg/g), mean ± SD	68.0 ± 35.5	85.3 ± 79.9	0.598
Hb (g/dL), mean ± SD	12.7 ± 0.8	13.0 ± 0.7	0.404
Ferritin (ng/mL), mean ± SD	24.2 ± 20.2	22.3 ± 16.7	0.827
BMI (kg/m^2^), mean ± SD	25.8 ± 4.0	27.6 ± 4.1	0.365

^a^ Mann–Whitney U test control (CTRL) vs. non-celiac wheat sensitivity (NCWS) group; ^b^ chi-squared test or Fisher’s exact test, when appropriate. Hp: *Helicobacter pylori*; TGA-IgA: transglutaminase immunoglobulin A; EMA: endomysial antibodies; ESR: erythrocyte sedimentation rate; CRP: C-reactive protein; Hb: haemoglobin; BMI: body mass index.

**Table 2:** Summary of differentially expressed (DE) transcripts in non-celiac wheat sensitivity (NCWS) and CTRL.

	Number of Transcripts Analysed	%	Absolute Mean Difference > 1	%	Benjamini–Hochberg Correction *p*-Value < 0.05	%
**Total**	**53,217**	**100**	429	100	**300**	**100**
**Protein-coding**	**27,883**	**52.4**	188	43.8	**110**	**36.7**
**Non-coding**	**25,334**	**47.6**	241	56.2	**190**	**63.3**
**Xloc or Loc**	**4769**	**18.8**	42	17.4	30	15. 8
**LNC**	**9200**	**36.3**	84	34.9	65	34.2
**Linc**	**1419**	**5.6**	17	7.1	17	8.9
**-AS**	**1465**	**5.8**	13	5.4	11	5.8
**SNORA-**	**118**	**0.46**	1	0.4	1	0.5
**SNORD-**	**185**	**0.73**	2	0.8	2	1.1
**-IT**	**63**	**0.24**	1	0.4	1	0.5
**No name**	**8115**	**32.0**	81	33.6	63	33.2

The first two columns report a classification and the number of transcripts analysed by microarray. The column labelled “Absolute Mean Difference > 1” shows the number of transcripts whose mean expression levels differed at least 1 unit between NCWS and control. The column labelled “Benjamini–Hochberg Correction *p*-value < 0.05” shows the number of transcripts whose mean expression levels differed at least 1 unit and were statistically different after false discovery rate (FDR) correction. The percentages of “protein-coding” and “non-coding” relate to the total number of transcripts. The percentage of Xloc, Loc, LNC, Linc, etc. relates to the number of non-coding transcripts.

**Table 3:** Area under the receiver operating characteristic (ROC) curve of the most relevant transcripts selected by LASSO regression model.

Probe Name	Gene Symbol	Variable Importance	AUC (95% CI)
A_22_P00006238	N/A	−2.299	0.990 (0.958–1.000)
A_21_P0011074	XLOC_l2_003253	1.795	0.969 (0.901–1.000)
A_21_P0005811	lnc-DUSP4-2	1.413	0.939 (0.838–1.000)
A_22_P00022828	lnc-RLBP1-1	−0.480	0.923 (0.806–1.000)
A_21_P0011522	XLOC_l2_005692	0.506	0.908 (0.784–1.000)
A_23_P27207	**SfigureCGB1C1**	−0.028	0.908 (0.773–1.000)
A_33_P3389286	**SFN**	0.263	0.913 (0.779–1.000)
A_24_P36229	**DTNA**	−0.105	0.893 (0.755–1.000)
A_21_P0001949	LOC101927431	0.881	0.867 (0.643–1.000)
A_33_P3266564	**KRTAP5**-6	0.644	0.857 (0.689–1.000)
A_21_P0008666	lnc-SH3GL3-1	−0.173	0.827 (0.568–1.000)
A_24_P263284	**CSMD2**	−0.325	0.770 (0.543–0.998)
A_24_P281395	N/A	−0.006	0.755 (0.462–1.000)
A_21_P0007206	lnc-CTD-2210P24.4.1-3	0.226	0.745 (0.478–1.000)
A_22_P00001191	lnc-ANGPTL2-3	−0.121	0.684 (0.391–0.977)

Each DE transcript is identified by a probe name and a gene symbol. Protein-coding transcripts are in bold. The variable importance values indicate the importance of a transcript as defined by the LASSO regression model. The area under the curve (AUC) indicate the probability of correctly classifying NCWS patients.

## Supplementary Materials

The following are available online at https://www.mdpi.com/1422-0067/21/6/1969/s1, Figure S1. Frequencies of gene expression differences between NCWS and controls. Figure S2. Heat map and PCA representation of the transcripts selected by LASSO. Figure S3. Intestinal malignancies sharing protein expression alterations with NCWS. Table S1. List of differentially expressed transcripts.

## Abbreviations

ATI	amylase trypsin inhibitor
AUC	area under the curve
DE	differentially expressed
FDR	false discovery rate
FODMAPs	fermentable oligo-, di-, monosaccharides
HLA	human leukocyte antigen
IBS	irritable bowel syndrome
IPA	ingenuity pathway analysis
LASSO	least absolute shrinkage and selection operator
lncRNA	long non-coding RNA
LPS	lipopolysaccharide
NCWS	non-celiac wheat sensitivity
PCA	principal component analysis
SIBO	small intestinal bacterial overgrowth
