# Supplementary figures and images for: An Exploratory Gene Expression Study of the Intestinal Mucosa of Patients with Non-Celiac Wheat Sensitivity
Source: Int J Mol Sci. 2020 Mar 13;21(6):1969. doi: 10.3390/ijms21061969 (PMC7139384; doi:10.3390/ijms21061969)

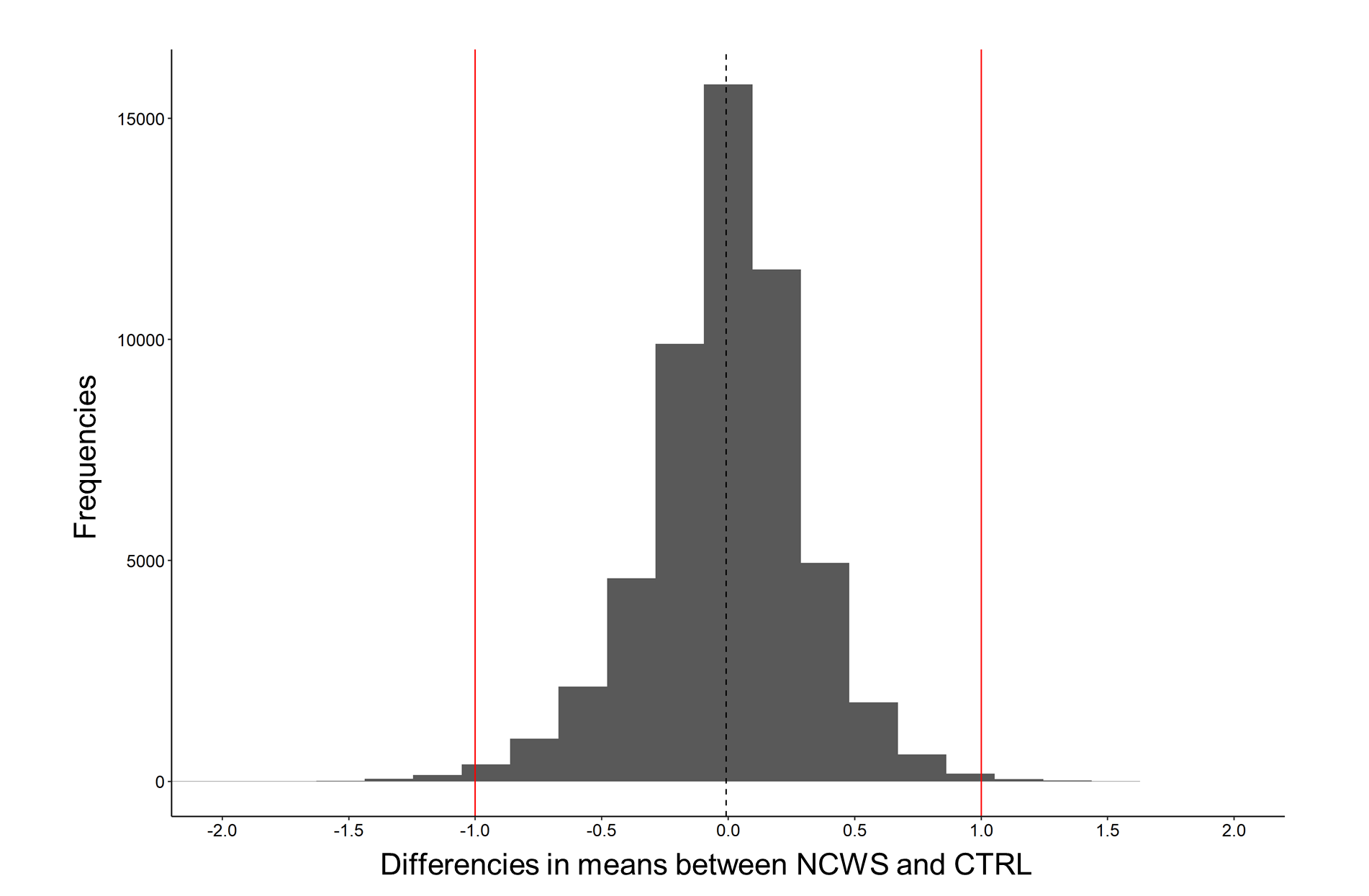

Supplement: Supplementary file 1 [file ijms-21-01969-s001.zip › SuppL_Fig1.tif]

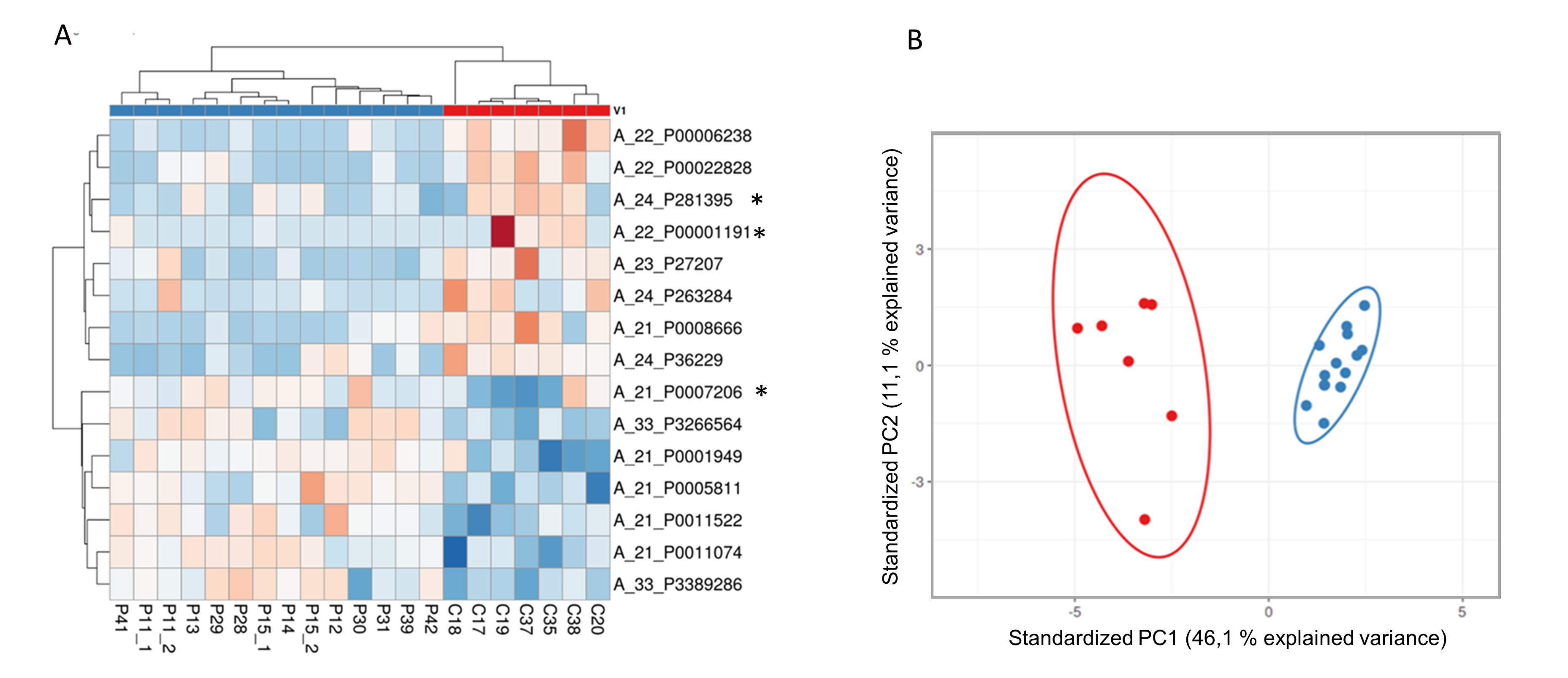

Supplement: Supplementary file 1 [file ijms-21-01969-s001.zip › Suppl_Fig2.tif]

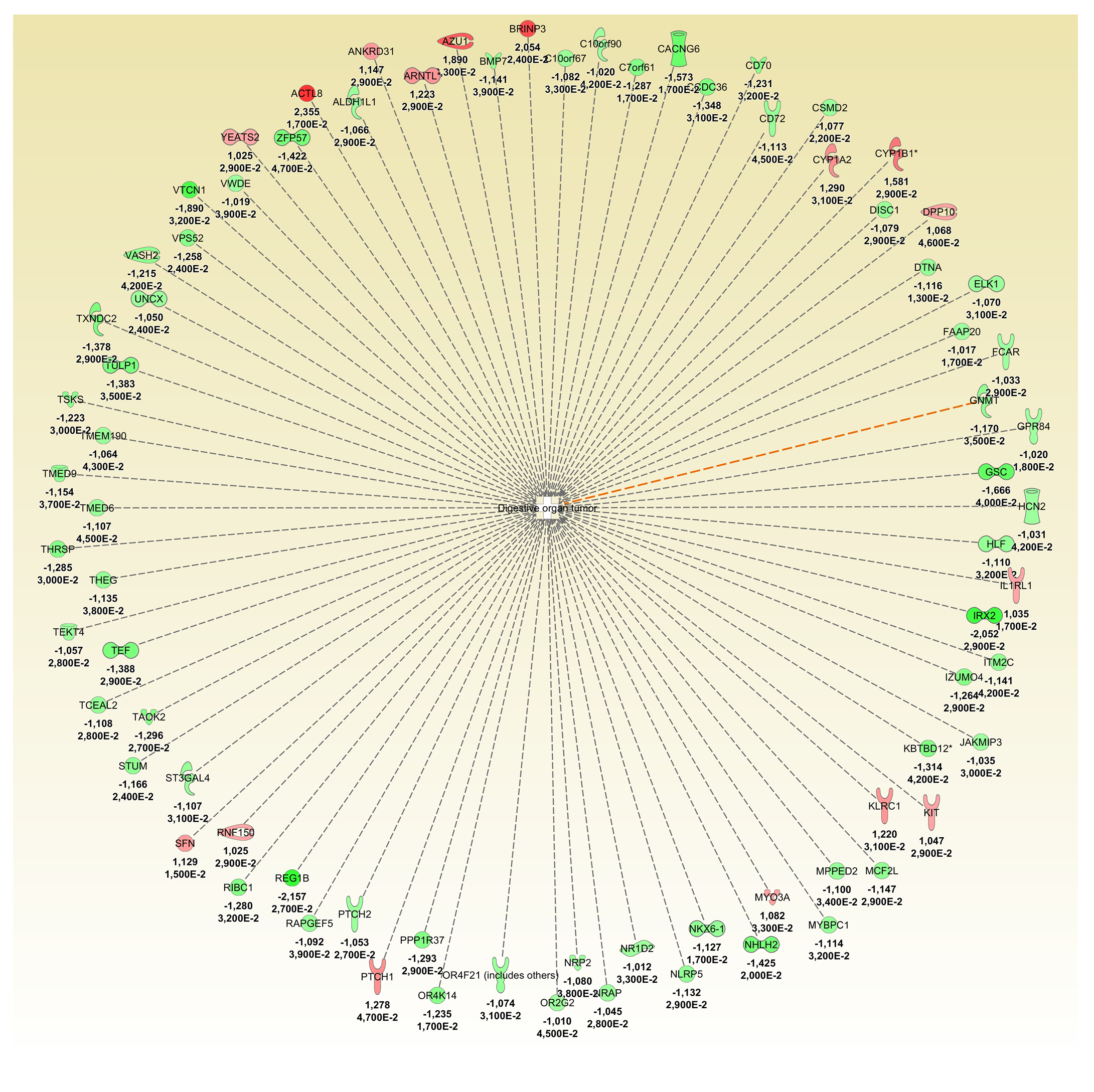

Supplement: Supplementary file 1 [file ijms-21-01969-s001.zip › Suppl_Fig3.tif]
